# Hepatic Arterial Infusion Chemotherapy for Advanced Hepatocellular Carcinoma in Japan

**DOI:** 10.3390/cancers4010165

**Published:** 2012-02-21

**Authors:** Hiroki Nishikawa, Yukio Osaki, Ryuichi Kita, Toru Kimura

**Affiliations:** Department of Gastroenterology and Hepatology, Osaka Red Cross Hospital, 5-30 Fudegasaki-cho, Tennoji-ku, Osaka 543-0027, Japan; E-Mails: yosk@osaka-med.jrc.or.jp (Y.O.); rkita@mvi.biglobe.ne.jp (R.K.); cxp00112@nifty.ne.jp (T.K.)

**Keywords:** advanced hepatocellular carcinoma, chemotherapy, hepatic arterial infusion chemotherapy, sorafenib, response rate

## Abstract

Transcatheter methods such as transcatheter arterial chemoembolization (TACE) and hepatic arterial infusion chemotherapy (HAIC) have an important role in the treatment for advanced hepatocellular carcinoma (HCC). Recently, sorafenib, an inhibitor of tyrosine kinases, has been found to obtain survival benefits in patients with HCC, leading to major advances in the treatment of advanced HCC. However, it is associated with a low tumor response rate, minimal survival advantage, and high rates of adverse events. On the other hand, high rates of objective treatment response with HAIC for advanced HCC have been reported, although convincing evidence of it contributing to overall survival in HAIC has been lacking. In Japan, HAIC still tends to be the preferred method for the treatment of advanced HCC, even in patients with poor liver function. However, the choice of chemotherapeutic agents in TACE/HAIC for HCC varies between institutions. In this review, based on studies reported to date in the literature, we refer to current knowledge regarding the chemotherapeutic agents used for TACE/HAIC for HCC in Japan and consider the future perspectives for HAIC for this cancer.

## 1. Introduction

Hepatocellular carcinoma (HCC) is a problem worldwide, especially in Asian countries [1,2,3] Unlike most solid cancers, the incidence and mortality rates for HCC are projected to increase substantially in many countries over the next 20 years, mostly as a result of viral infections with hepatitis C and hepatitis B [4]. It has become possible to identify a group of patients with chronic liver disease who are at a high risk of developing HCC. In addition, improvements in diagnostic imaging have allowed early diagnosis of HCC. However, unfortunately, most HCC patients are first seen when the disease has reached an advanced stage at which curative treatment is no longer possible [4].

Potentially curative treatments for HCC include hepatic resection and liver transplantation [5,6]. Several non-surgical treatment options, including percutaneous ethanol injection, microwave coagulation, and radiofrequency ablation (RFA) have also been developed and are widely used for the treatment of HCC [4]. However, in general, these modalities are not indicated for patients with multiple tumors, invasion or thrombosis of major vessels, extrahepatic metastases, or poor liver function. Various anticancer agents have been employed for transcatheter arterial chemoembolization (TACE) and hepatic arterial infusion chemotherapy (HAIC) in these patients with advanced HCC, in whom effective chemotherapy is required.

Regarding systemic chemotherapy for advanced HCC, no agent has reproducibly shown a high response rate, and few chemotherapeutic regimens have a meaningful impact on patient survival, although numerous clinical trials have been performed to establish effective systemic treatment for patients with advanced HCC [7,8,9,10,11].

Sorafenib, an inhibitor of tyrosine kinases, was the first systemic chemotherapeutic agent found to improve the survival time of patients with advanced HCC, in the SHARP trial [12]. However, it is associated with a low tumor response rate, minimal survival advantage, and high rates of adverse events [12]. Moreover, because most patients enrolled in this study had Child–Pugh A cirrhosis with well-preserved liver function, the benefits and safety profile of sorafenib in advanced HCC patients with poor liver function such as Child–Pugh B/C cirrhosis or other poor prognostic factors remain unclear. Alternative approaches to the treatment of advanced HCC are needed, especially in patients with poor liver function. 

Transcatheter arterial iodized oil chemoembolization has become established as an effective treatment for unresectable HCC. However, it is generally suitable only for patients with well-preserved liver function and multiple tumors without major vascular invasion [13,14]. HAIC is one of the few remaining options for advanced HCC patients with poor liver function [15]. In general, patients with HCC are less tolerant to chemotherapy than patients with other malignancies owing to poor liver function. In most HCC patients, pancytopenia may already be present because of concomitant liver cirrhosis, and myelosuppression and a bleeding tendency are likely to occur when performing chemotherapy. Owing to these clinical features of cirrhotic patients, HAIC is not commonly used for HCC in North America and Europe. However, in cases in which extrahepatic spread is minimal and local control of liver tumors is considered more important, HAIC is useful and may offer survival benefits, even when there are extrahepatic metastases. HAIC has often been used in these cases in Japan [16,17].

Compared with systemic chemotherapy, HAIC requires several technical procedures, including catheterization, and is associated with a risk of vascular disorders related to catheter placement and reservoir management [17]. However, it is a useful therapeutic modality because higher concentrations of the anticancer agent are obtained in the targeted tumor. The agent is administered directly into the liver via the hepatic artery, increasing the antitumor effect while being associated with a lower rate of incidence of systemic adverse events, and numerous clinical studies have reported that HAIC has moderate chemotherapeutic effect with favorable toxicity profiles in selected patients with advanced HCC [17]. However, there have been no large randomized studies and convincing clinical evidence is lacking for HAIC in advanced HCC. Therefore, HAIC is not yet a well-established treatment for advanced HCC, and its further investigation is required. 

Several intra-arterial chemotherapy regimens, using doxorubicin, epirubicin, mitomycin C, 5-fluorouracil (5-FU), zinostatin stimalamer (SMANCS), cisplatin, miriplatin and oxaliplatin administered singly or in combination, have been reported as treatments for HCC. However, the optimal regimen for intra-arterial chemotherapy for HCC remains unknown. In this review, based primarily on clinical studies reported to date in the literature regarding transcatheter chemotherapeutic agents in HAIC for advanced HCC, we refer to current knowledge regarding the chemotherapeutic agents used in Japan for HCC and consider the future perspectives for hepatic arterial chemotherapy for this cancer.

## 2. Chemotherapeutic Agents

### 2.1. Doxorubicin

Doxorubicin (Adriacin; Kyowa Hakko Kogyo, Tokyo, Japan) is an anthracycline-based anticancer drug that had been conventionally used as a first-line therapy for HCC. In 1982, Hirose *et al.* reported that one shot of high-dose doxorubicin intra-arterial infusion therapy showed useful chemotherapeutic effects against HCC [18]. In 1994, Yasui *et al.* reported that of 86 patients with unresectable or recurrent HCC who underwent doxorubicin containing HAIC, 21 (34.4%) showed an objective response, and responders to doxorubicin containing HAIC achieved longer survival than non-responders [19]. In 1999, Tzoracoleftherakis *et al.* reported a randomized comparative study between HAIC with doxorubicin and systemic chemotherapy with doxorubicin involving 72 patients with unresectable (Stage IVA) HCC; patients who received HAIC with doxorubicin had a higher rate of objective and subjective remission and greater Karnofsky performance status improvement than did those in the systemic chemotherapy with doxorubicin group [20]. However, previous studies of systemic chemotherapy with doxorubicin had confirmed the minimal efficacy of this anticancer drug in HCC [9,10,11]. In Japan, doxorubicin is used for both HAIC and TACE in the treatment of advanced HCC [21].

### 2.2. Epirubicin

Epirubicin (Farmorubicin; Nihon Kayaku, Tokyo, Japan) is the 4-epimer of doxorubicin. It has a more favorable toxicity profile with less myelosuppression and cardiotoxicity than doxorubicin, and is routinely used for HCC. Because epirubicin easily undergoes glucuronidation, it is less toxic than doxorubicin. In 1986, the Japan Epirubicin Study Group for Hepatocellular Carcinoma reported that of 53 HCC patients who received HAIC with epirubicin, eight patients (15.1%) showed an objective response (complete response [CR] or partial response [PR]) [22]. Furthermore, a retrospective comparison with intra-arterial administration of doxorubicin showed that epirubicin was more effective than doxorubicin in terms of survival rate. Since then, epirubicin has been routinely used in HAIC for HCC in Japan [22]. Additional clinical trials have been performed in several faculties. Epirubicin alone or in combination with other chemotherapeutic agents such as mitomycin C or 5-FU has been used in HAIC for HCC in various Asian countries including Japan, with objective response rates ranging from 5% to 78% [22,23,24,25,26,27,28,29]. Reports of HAIC with anthracycline-based chemotherapeutic agents are listed in Table 1.

**Table 1:** Reports of HAIC with anthracycline-based chemotherapeutic agents for advanced hepatocellular carcinoma.

Authors (year) [ref.]	Country	Number of patients	Agents	Characteristics	Response rate (%)
Nagasue *et al.* (1986) [22]	Japan	53	Epirubicin	Unresectable	15.1
Yoshikawa *et al.* (1994) [23]	Japan	17	Epirubicin	Unresectable	12
Yasui *et al.* (1994) [19]	Japan	86	Doxorubicin, MMC, 5-FU	Unresectable	34.4
Tzoracoleftherakis *et al.* (1999) [20]	Greece	72	Doxorubicin	Unresectable	60
Hwang *et al.* (2005) [24]	Korea	18	Epirubicin, MMC, 5-FU	Unresectable	38.9
Ikeda *et al.* (2007) [25]	Japan	45	Epirubicin	PV invasion	9
Tanaka *et al.* (2008) [26]	Japan	20	Epirubicin	TAE refractory	5
Ikushima *et al.* (2009) [27]	Japan	18	Epirubicin	Unresectable	77.8
Kim *et al.* (2010) [30]	Korea	36	Doxorubicin	Unresectable	16.7

HAIC: hepatic arterial infusion chemotherapy; MMC: mitomycin C; 5-FU: 5-fluorouracil; PV: portal vein; TAE: transcatheter arterial embolization.

### 2.3. Mitomycin C

Mitomycin C is an antineoplastic antibiotic isolated from the culture fluid of *Streptomyces caespitosus*. The objective response rate of intra-arterial chemotherapy with mitomycin C alone has been reported as 25% (9/36) [31]. However, this agent has recently been used in HAIC as a combination therapeutic agent. In Japan, mitomycin C alone has never been used in HAIC [21,24,32].

### 2.4. 5-FU

The pyrimidine antimetabolite 5-FU was the first reported chemotherapeutic agent used in the treatment of HCC. Monotherapy with intra-arterial 5-FU has a relatively low objective response rate, ranging from 13% to 22%, with a median survival of only 3.5 to 14 months [33,34]; therefore, 5-FU is often used in combination in HAIC for HCC.

Combined therapy comprising intra-arterial infusion of 5-FU and systemic interferon-α (IFN-α) (FAIT) has been reported to be useful as a palliative treatment for HCC patients with major vascular invasion, although monotherapy with IFN-α had minimal objective response rate against HCC [35,36,37]. In 2002, Sakon *et al.* reported that of 11 advanced HCC patients with portal vein tumor thrombus (PVTT), eight patients (73%) showed an objective response to FAIT [38]. In 2006, Obi *et al.* reported that in 116 advanced HCC patients with PVTT who received FAIT, 19 (16%) showed a CR and another 42 (36%) showed a PR; adverse events were limited to nausea and appetite loss, and the overall survival rates at 1 and 2 years were 34% and 18%, respectively [39]. In 2011, Nagano *et al.* reported that of 102 HCC patients with PVTT, 40 (39.2%) showed an objective response to FAIT, and no major treatment-related complications were noted [40]. In 2006, Kondo *et al.* reported that a combination of 5-FU and IFN-α strongly inhibited the growth of human HCC cells, and suggested that the effects of this combination therapy may be attributable to changes in the induction of apoptosis through IFN-α/β receptors [41]. In 2009, Kasai *et al.* reported that intra-arterial 5-FU and systemic pegylated (PEG)-IFN-α2b combination therapy for advanced HCC had an objective response rate of 71.4% [42]. In 1999, Yano *et al.* compared the *in vivo* antitumor effects of PEG-IFN-α2b and IFN-α in nude mice injected with cultured HCC cells, and found that PEG-IFN-α2b induced apoptosis more strongly than IFN-α [43]. Intra-arterial 5-FU infusion and systemic PEG-IFN-α2b combination therapy appears to be highly promising, although further prospective studies are required [42,44]. Reports of the use of HAIC with 5-FU combined with systemic IFN in advanced HCC are listed in Table 2.

**Table 2:** Reports of hepatic arterial infusion chemotherapy with 5-FU combined with systemic interferon for advanced hepatocellular carcinoma.

Authors (year) [ref.]	Country	Number of patients	Characteristics	IFN	Response rate (%)
Sakon *et al.* (2002) [38]	Japan	8	PV invasion	IFN-α	62.5
Enjoji *et al.* (2005) [45]	Japan	28	PV invasion or unresectable	IFN-α	21.5
Ota *et al.* (2005) [46]	Japan	55	PV invasion	IFN-α	43.6
Obi *et al.* (2006) [39]	Japan	116	PV invasion	IFN-α	52.5
Uka *et al.* (2007) [47]	Japan	31	PV invasion	IFN-α	29.1
Kuroda *et al.* (2007) [48]	Japan	10	PV invasion	IFN-α	10
Katamura *et al.* (2009) [49]	Japan	16	PV invasion	IFN-α	25
Kasai *et al.* (2009) [44]	Japan	9	PV invasion	Peg-IFN α2b	77.8
Kasai *et al.* (2011) [42]	Japan	21	PV invasion	Peg-IFN α2b	71.4
Nagano *et al.* (2011) [40]	Japan	102	PV invasion	IFN-α	39.2

IFN: interferon; PV: portal vein; Peg-IFN: pegylated interferon.

Low-dose cisplatin (CDDP) combined with 5-FU (low-dose FP therapy) has synergistic effects. This combination is often used in the treatment of gastrointestinal tract malignancies. In combination with 5-FU, cisplatin is a modulator rather than an effector, and increases the antitumor efficacy of 5-FU by increasing the intracellular concentration of reduced folate [37,50]. In 2002, Ando *et al.* reported that of 48 HCC patients with PVTT, 23 (48.0%) showed an objective response to low-dose FP therapy and concluded that HAIC using low-dose FP might be a useful therapeutic option for patients with advanced HCC with PVTT [51]. In 2010, Ueshima *et al.* reported that of 52 advanced HCC patients, 20 (38.5%) showed an objective response to low-dose FP therapy [52]. However, there are few reports of favorable objective response rates with the use of high-dose CDDP combined with 5-FU therapy for the treatment of advanced HCC [53,54,55]. Reports of intra-arterial 5-FU and CDDP combination therapy in HAIC are listed in Table 3.

**Table 3:** Reports of hepatic arterial infusion chemotherapy with 5-FU and CDDP for advanced hepatocellular carcinoma.

Author (year) [ref.]	Country	Number of patients	Characteristics	Dose	Response rate (%)
Ando *et al.* (2002) [51]	Japan	48	PV invasion	Low	48
Itamoto *et al.* (2002) [56]	Japan	7	PV invasion	Low	33
Lai *et al.* (2003) [57]	China	18	PV invasion	Low	33
Sumie *et al.* (2003) [58]	Japan	16	PV invasion	Low	56.3
Yamasaki *et al.* (2005) [59]	Japan	15	Unresectable	Low	20
Park *et al.* (2007) [54]	Korea	41	Unresectable	High	22
Kim *et al.* (2010) [53]	Korea	36	Unresectable	High	16.7
Ueshima *et al.* (2010) [52]	Japan	52	PV invasion or unresectable	Low	38.5
Woo *et al.* (2010) [55]	Korea	32	PV invasion	Low	0
Woo *et al.* (2010) [55]	Korea	36	PV invasion	High	16.7

PV: portal vein.

### 2.5. CDDP

Clinical investigation of cisplatin (*cis*-diamminedichloroplatinum; CDDP) had been started in 1972. The usefulness of CDDP as an anticancer agent was first confirmed in the treatment of urinary tract cancers. Currently, CDDP is a key chemotherapeutic agent for the treatment of various cancers, including tumors of the respiratory, genitourinary, and digestive systems [60]. The anticancer effect of CDDP is characterized by both time-dependent and concentration-dependent features.

The response rate of CDDP monotherapy administered by HAIC for advanced HCC ranges from 14% to 42% [61,62,63,64]. Reported objective response rates to arterial infusion regimens containing CDDP, such as low-dose FP therapy, range from 0% to 56%, although high efficacy of systemic chemotherapy with CDDP has not been reported in HCC [17,51,52,53,65,66,67,68,69,70,71]. 

Microfine powder CDDP preparations (DDP-H) (IA-call; Nippon Kayaku Co., Ltd., Japan) for arterial infusion were approved for its use in Japan in 2004. Recently in Japan, favorable results have been obtained using DDP-H in the treatment of advanced HCC patients. In 2008, Yoshikawa *et al.* conducted a phase II study in advanced HCC patients, reporting that in 80 patients who received HAIC with DDP-H, the overall response rate was 33.8%, the 1-year and the 2-year survival rates were 67.5% and 50.8%; respectively, they concluded that DDP-H has higher antitumor effect than other anticancer drugs when administered by HAIC [62]. The usefulness of DDP-H as a second-line treatment for advanced HCC refractory to TACE using an epirubicin-lipiodol emulsion has also been reported [72,73].

Systemic combination therapy with S-1 and CDDP is a promising treatment for advanced HCC. In 2010, Katamura *et al.* reported that of 16 HCC patients with extrahepatic metastases who received S-1 and CDDP combination therapy, two (13%), none (0%), five (31%), and nine (56%) showed CR, PR, stable disease, and progressive disease, respectively, with an overall objective response rate of 13% (2/16) and an overall survival rate at 1 year of 77% [74].

There is one report that RFA with sequential HAIC using cisplatin contributed to a longer disease-free interval [16]. HAIC using cisplatin before RFA might prevent an increase in the size of pre-existing microscopic tumor foci.

### 2.6. SMANCS

SMANCS is a lipophilic intra-arterial chemotherapeutic agent for HCC. In 1983, Konno *et al.* reported a reduction in tumor size and tumor markers in 13 of 14 patients with HCC following arterial infusion of SMANCS-lipiodol emulsion [75]. A subsequent study of 124 patients with HCC or metastatic liver tumors showed significantly higher survival rate following infusion of a SMANCS-lipiodol emulsion [76].

In 2002, Okusaka *et al.* reported a phase II study of TACE using SMANCS in which the overall response rate was 32% (16/50); they concluded that TACE using SMANCS, which was well tolerated, might be a useful treatment for advanced HCC [77]. However, hepatic arterial infusion with SMANCS caused severe vascular endothelial damage and loss of the hepatic artery for infusion [77,78]. SMANCS is thus unsuitable for repeated TACE/HAIC in HCC and is not widely used for this purpose in Japan.

### 2.7. Miriplatin

Miriplatin (Miripla; Dainippon Sumitomo Co., Ltd., Tokyo, Japan), a cisplatin derivative, is a novel chemotherapeutic agent designed for use in transarterial infusion chemotherapy for HCC [79]. Miriplatin: (1) inhibits cell proliferation in a similar manner to cisplatin and has superior solubility in ethyl esters of iodized fatty acids derived from poppy seed oil; (2) releases its platinum constituent continuously, together with the ethyl esters (sustained release), by remaining at the site of the tumor; and (3) has fewer adverse effects, because of its sustained release and its minimal presence in the general circulation [80,81,82,83]. In Japan, miriplatin was approved for use in October 2009.

In 2004, Okusaka *et al.* conducted a phase II study of miriplatin to assess its antitumor effect and toxicity in treatment-naive patients with HCC. They reported that the CR rate was 56% (9/16) and that none of the patients exhibited grade 4 toxicity or episodes of renal dysfunction, and concluded that miriplatin was well tolerated and showed promising antitumor effect in patients with HCC [84]. In 2011, Okusaka *et al.* reported their phase II comparative study of miriplatin and SMANCS, in which HAIC with miriplatin had a similar efficacy to HAIC with SMANCS, and repeated dosing with miriplatin was possible without hepatic vascular injury in cases of relapse, whereas HAIC with SMANCS caused hepatic vascular injury [78]. In 2011, Imai *et al.* reported that in 162 unresectable HCC patients who underwent transcatheter arterial chemotherapy using miriplatin with or without embolization, the objective response rates to HAIC with miriplatin and TACE with miriplatin were 33% (13/40) and 57% (70/120), respectively; they concluded that an objective response was achieved in a significantly higher number of patients treated with TACE with miriplatin than with HAIC with miriplatin [85].

It is well known that HAIC with CDDP causes renal dysfunction [86]. However, in 2011, Imai *et al.* reported that HAIC with miriplatin could be used safely in HCC patients with chronic renal failure, probably owing to its minimal presence in the general circulation [87]. In terms of renal toxicity, HAIC with miriplatin is considered safer than HAIC with CDDP.

In an animal experiment comparing the antitumor effects of two platinum agents (miriplatin and DDP-H), Watanabe *et al.* reported no significant difference between miriplatin-lipiodol emulsion and DDP-H-lipiodol emulsion after 7 days post-administration [88]. To date, however, there is no convincing clinical evidence regarding the antitumor effects of these drugs. In Japan, several clinical studies comparing the antitumor effects of miriplatin and other chemotherapeutic agents such as CDDP and epirubicin for advanced HCC are ongoing. If the results for miriplatin are positive in these studies, it will become a key chemotherapeutic drug in the treatment of unresectable HCC.

### 2.8. Oxaliplatin

The platinum-based chemotherapeutic agent oxaliplatin (L-OHP) displays a wide range of antitumor activities [89]. Oxaliplatin has often been used for the treatment of advanced colorectal cancer, in which its effectiveness has been confirmed in many clinical studies [90]. To date, however, few detailed clinical data are available concerning the effects of HAIC with oxaliplatin in advanced HCC. In Japan, oxaliplatin has not been used for the treatment of advanced HCC. In 2010, Rathore *et al.* reported that in their phase I trial, HAIC with oxaliplatin was feasible, well tolerated, and had demonstrated activity in patients with advanced HCC [91]. A phase II prospective randomized study of the effectiveness of HAIC with oxaliplatin in unresectable HCC is underway. If positive results are obtained, oxaliplatin could become a key chemotherapeutic agent in the treatment of advanced HCC.

## 3. Discussion

There are four main reasons why HAIC may be well suitable for the treatment of advanced HCC compared with systemic chemotherapy. First, because HCC derives almost all of its blood supply from the hepatic artery, high anticancer drug concentrations can be obtained while hepatic perfusion is maintained via the portal vein [17,92,93]. Second, normal hepatic tissue metabolizes various agents, such that first pass metabolism leads to higher local drug concentrations in the liver, reducing systemic adverse effects [17,93]. Third, prolonged drug exposure, which may increase the antitumor effect, is easily obtained with HAIC [17,93]. Fourth, unlike other locoregional therapies for HCC, HAIC is not limited by tumor size, tumor number, or proximity to major vascular structures, all of which preclude surgical resection or RFA in general. 

There are two methods for administration of HAIC: one-shot hepatic arterial infusion and continuous hepatic arterial infusion. In one-shot hepatic arterial infusion, concentration-dependent agents such as anthracycline-based agents, mitomycin C, CDDP, and miriplatin are suitable for HAIC. In continuous hepatic arterial infusion, 5-FU, which exerts its antitumor effects time-dependently, anthracycline-based agents, mitomycin C, and intermittent administration of CDDP based on the concept of biochemical modulation are suitable for HAIC. In continuous hepatic arterial infusion, an implantable port system is required. Several recent studies indicate that probably owing to technical improvements of angiographic procedure, only 0–4% of patients develop catheter-related complications such as breakage of the reservoir device, catheter dislocation, artery dissection, artery occlusion, subcutaneous hematoma, and infection [38,56]. Therefore, HAIC using an implantable port system can be performed safely.

In general, HCC responds poorly to chemotherapy. The possible explanations are tumor heterogeneity, inducible overexpression of the multidrug resistance gene, and/or inherent resistance due to an unknown mechanism [94,95,96,97]. Combination therapy is therefore considered to be more effective than monotherapy. Synergistic cooperative effects have been observed in experiments on HCC cell lines [96].

In sorafenib treatment for advanced HCC, as mentioned above, because most patients enrolled in the SHARP trial had Child–Pugh A cirrhosis with well-preserved liver function, the benefits and safety profile of sorafenib in advanced HCC patients with Child–Pugh B/C or other poor prognostic factors remains unclear [12]. However, even patients with poor performance status or reduced hepatic functional reserve could be eligible for HAIC. Indeed, as shown in Table 2, Table 3, many studies of HAIC have focused on HCC patients with PVTT. Moreover, the objective response rate of sorafenib treatment is low in general. In the SHARP trial, it was very low (CR and PR were 0% and 2%, respectively) [12]. In Japan, however, there have been many reports of high rates of response to HAIC in advanced HCC, although most of these studies were not randomized and few demonstrated any survival benefit using controls. The clinical response reflects the survival benefits [14,37,38,39,52,98,99]. Probably, chemosensitive subgroups of patients with advanced HCC are present, and it is therefore important to identify optimal candidates for HAIC as well as to investigate novel therapeutic strategies. A novel method using gene expression profiling has recently been reported for the prediction of treatment response in advanced HCC [100]. In practice, however, responders and non-responders should be distinguishable after the first session of HAIC by evaluating tumor size using imaging modalities and the levels of tumor markers. In early non-responders, HAIC should not be continued; instead, different therapeutic options, including sorafenib, should be explored. We have experience of a case of advanced HCC with PVTT refractory to epirubicin that showed a marked decrease in tumor markers after HAIC with miriplatin [101].

## 4. Future Perspectives

The favorable objective response rate reported for HAIC in advanced HCC in Japan provides a sound basis for its clinical use. In the era of molecular targeted anticancer therapies such as sorafenib, incorporating such agents into HAIC regimens may obtain further improvement of treatment effect. However, the effects of combination therapy of sorafenib and HAIC are unclear. According to a subgroup analysis of the SHARP trial, it is speculated that sorafenib in combination with resection, ablation, TACE or HAIC will prolong overall survival in various stages of HCC [12,102]. There are several case reports showing a favorable response of advanced HCC to combination therapy with sorafenib and HAIC [103,104], although in 2011 Kemeny *et al.* reported that in 22 primary liver cancer patients, addition of systemic bevacizumab to HAI floxuridine/dexamethasone appeared to increase biliary toxicity without any clear improvement in outcome [105]. To elucidate this issue, a randomized clinical trial of sorafenib alone versus sorafenib combined with maintenance TACE/HAIC and/or HAIC in intermediate and advanced HCC was initiated in Japan in 2009 [102]. If favorable results are obtained by these trials, the treatment strategy for HCC will be drastically changed.

## 5. Conclusions

As shown in Table 1, Table 2, Table 3, many clinical studies with favorable response rates for HAIC in advanced HCC have been reported in Japan, and the improvement of survival can be ascribed to treatment-related effects. We believe that HAIC as well as sorafenib should be considered an effective treatment for advanced HCC.
